# Quantitative Assessment of the Effects of Scatter Radiation on Image Quality During Extremity Radiography at a Leading Tertiary Hospital in Ghana: An Experimental Pilot Study

**DOI:** 10.1002/hsr2.72353

**Published:** 2026-04-16

**Authors:** Erica Mawusinu Domi, Jacob Leonard Ago, William K. Antwi, Derick Seyram Sule, Godwill Acquah, Godsway Yao Gagoh, Ophelia Andowah Otsiwah

**Affiliations:** ^1^ Department of Radiography, School of Biomedical and Allied Health Sciences University of Ghana Accra Ghana; ^2^ Discipline of Medical Radiations, School of Health and Biomedical Sciences RMIT University, Bundoora Campus Melbourne Victoria Australia; ^3^ Radiography Department Medi Moses Diagnosis Centre Accra Ghana

**Keywords:** dividing receptor technique, extremity radiography, image quality, scatter radiation

## Abstract

**Background and Aims:**

There is a potential increase in scatter radiation during extremity radiography, where radiographers may divide the detector to obtain multiple images on one film. This study assesses the impact of scatter radiation on image quality (IQ) during the dividing receptor technique in extremity radiography at the Korle‐Bu Teaching Hospital, Ghana.

**Methods:**

An experimental study design was employed. The examinations were performed with a computed radiography system from June 6, 2024, to July 4, 2024, using a dividing receptor technique. Scatter radiation was evaluated as whole‐body equivalent dose and surface dose at depths of 10 mm [H_p_(10)] and 0.07 mm [H_p_(0.07)], respectively. IQ was assessed using ImageJ software v1.54c. Data were analyzed descriptively and inferentially with IBM SPSS v29.0. A *p*‐value < 0.05 was considered statistically significant.

**Results:**

The measured scatter doses at H_p_(10) and H_p_(0.07) for all the examinations were 0.017 ± 0.0015 mSv and 0.0016 ± 0.0020 mSv, respectively. Whereas no statistically significant differences in signal‐to‐noise ratio (*p* = 0.53) were found for examinations performed before and after dividing the receptor, the difference in noise was found to be statistically significant (*p* < 0.001). Different anatomical regions generated varying, but non‐significant (*p* > 0.05), scatter radiations, noise, and signal‐to‐noise ratios. Pearson correlation tests revealed no statistically significant relationship between the reduced IQ and the measured H_p_(10) (*p* = 0.98) and H_p_(0.07) (*p* = 0.85).

**Conclusion:**

The study identified significant differences in noise and non‐significant differences in SNR, although the differences did not result from the scatter dose produced from the dividing receptor technique during extremity radiography. Nonetheless, there is a need for radiographers to adopt strategies to reduce the scatter radiation when using the dividing receptor technique. Consequently, we recommend radiation dose optimization techniques such as effective collimation and protocol adjustments during extremity radiography.

**Abbreviations**
ANOVA: analysis of variance
DRT: dividing receptor technique
IQ: image quality
IR: image receptor
SNR: signal‐to‐noise ratio
SPSS: Statistical Package for Social Sciences
TL: thermoluminescent
TLD: thermoluminescent dosimeter

## 1 Introduction

General radiographic examinations are the most used medical imaging techniques for diagnosing several pathologic conditions globally [1]. The physical principles of radiographic imaging involve (i) absorption, a phenomenon where the X‐rays interact with the patient's body and the energy is totally absorbed in the body; (ii) penetration, a phenomenon where the X‐ray traverses the human body without interaction; and/or (iii) scattering, where the X‐rays interact with the body and then scatter from its original trajectory and there may be loss or no loss of energy [2, 3, 4]. Scatter radiation may be classified as forward, back, or side scattering. Side scatter and backscatter radiation mainly affect the exposure dose to people around the imaging space (including patients, public, and personnel), while forward‐scattered X‐rays affect the recorded image [5, 6, 7].

Indeed, scatter radiation is a significant radiographic IQ reduction factor due to its presence in the final image [3]. Of note, the outcomes from any imaging technique depend on both image quality (IQ) and patient safety (including radiation dose). As a result, scatter radiation is regarded as an unacceptable phenomenon in radiography [1] as its effects on radiographic IQ (e.g., noise) can potentially hinder diagnoses and clinical management of diseases [8]. The amount of scattering is influenced by many factors such as X‐ray beam area, photon energy, tissue composition, and tissue thickness [1].

Scatter radiation may degrade IQ due to increased image noise and decreased radiographic contrast [1]. Radiographs considered to be of low quality due to unsatisfactory technical quality [9] are usually rejected, and the procedure is subsequently repeated [10]. In contravention of the optimization principle, this phenomenon of reject‐retake is of economic disadvantage as both human and radiological resources are further reduced [11]. Tellingly, it subjects patients and personnel to unnecessary exposure to ionizing radiation [12], which, no matter how minimal, has the possibility of increasing stochastic health risks.

Among the many causes of reject‐repeat radiographs for low‐quality images, scatter radiation manifesting as increased noise is considered the common indicator [13]. The effect of scatter radiation on IQ may be more predominant in radiographic examinations where bucky is less used, such as extremity radiography, that is, radiographic examination of the upper and lower limbs. This radiographic technique is often utilized in the context of trauma to rule out fractures and dislocations [14]. According to Chen [15], although some scattered radiation, such as that reaching the detector, can be minimized, it is impossible to completely eliminate them. However, various control measures may have a counter effect on the IQ [15]; thus, a comprehensive and balanced consideration should be made when choosing a correctional measure, which includes appropriate adjustment of technique factors and the use of protective lead plates [15].

In many imaging departments in Ghana, including the center where this study was conducted, extremity radiography is performed using a dividing receptor technique (DRT). In this technique, the first projection, usually the anteroposterior or posteroanterior view, is taken on one half of the detector/receptor, while the second projection, usually the lateral or oblique view, is taken on the other half of the detector/receptor. However, as these techniques are often performed without bucky, there are often no anti‐scatter grids [1], and in the case of Ghana, lead protections are seldom used, resulting in a compromised IQ [16] and increased radiation risk. Nonetheless, little is known about the extent of these effects, quantitatively. Therefore, this experimental study was performed to quantitatively assess the amount of scatter radiation produced during these radiographic techniques and their impact on IQ.

**Research questions**

1. How much scatter radiation is produced during DRT in extremity radiography?
2. What is the effect of the measured scatter radiation on IQ?

## 2 Materials and Methods

### 2.1 Study Design, Population, and Sample Size

An experimental design was adopted as they are ideal for studies requiring precise control and quantitative assessment of variables [17]. The study was conducted with patients referred for extremity radiographic examinations at the Accident and Emergency unit of the Korle‐Bu Teaching Hospital, Accra, Ghana, from June 6, 2024, to July 4, 2024. Yamane's [18] formula (Equation 1) was used to determine the sample size.

(1) $$n=\frac{N}{1+Ne^{2}}.$$

From Equation (1), *n* = sample size; *N* = population size; *e* = error margin. Data gathered from the study center revealed an estimated population size of 190 for a 1‐month duration. Assuming *e* = 0.05, the estimated *n* was 74. However, due to the nature of data collection, only 45 examinations were recorded, which constituted the final sample size.

### 2.2 Sampling Technique, Inclusion, and Exclusion Criteria

A systematic random sampling method was employed to select participants from the daily pool of patients undergoing extremity radiographic examinations during the study period. This was done to improve workflow as data was collected concurrently with routine radiographic examinations. Only patients between the ages of 18 and 80, inclusive, undergoing extremity radiographic examinations at the designated study centers who consented to participate were included. Non‐consenting patients and patients undergoing other radiographic procedures were excluded from this study.

### 2.3 Data Collection Tools

The main data collection tools were Shimadzu X‐ray equipment and thermoluminescent dosimeter (TLD) badges. In addition, a self‐designed Microsoft Excel spreadsheet was used to record patient details and exposure factors (Supporting Information: Sheet S1).

### 2.4 Reliability and Validity of Quantitative Data Collection Tools

Quality control tests were performed on the various X‐ray equipment prior to data collection. The tests included radiation and light‐field congruence (Supporting Information: Sheet S2), as well as an X‐ray beam alignment test and a physical inspection to make sure each piece of equipment was in good shape. Additionally, the TLD badges were kept enclosed in radiopaque containers to prevent ambient radiation from affecting the results of the study. Efforts were made to ensure that irradiated TLD badges were not reused until they were read. All these measures ensured the validity and reliability of the results.

### 2.5 Data Collection Process

#### 2.5.1 X‐Ray Examinations

**Figure 1:**
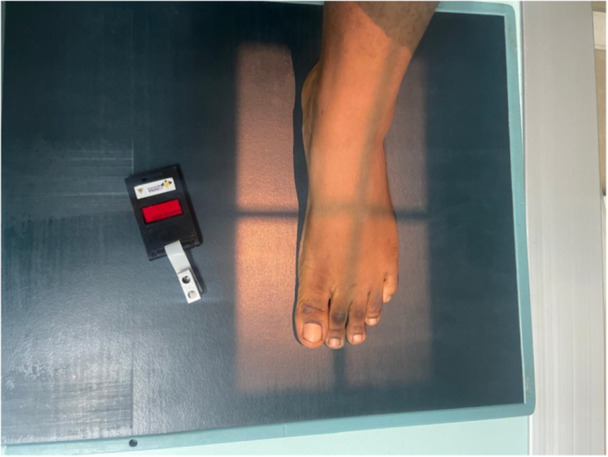
Data collection procedure.

For each examination, the detector was divided into two equal parts to accommodate two projections, after which the patient was positioned for an extremity radiographic examination. The TLD badge was then carefully positioned at the unexposed area, specifically at the side where the second projection was taken (Figure 1). Instructions were given to the patient to maintain that position until the procedure was completed. The X‐ray beam was collimated to cover only the area of interest, that is, the body part under examination. Appropriate exposure factors were then selected for the extremity under examination, and an exposure was made. After the image had been obtained, the TLD badge was carefully removed and stored in an airtight container to avoid direct exposure to sun rays, as exposure to sunlight can cause the readings to change, leading to inaccurate readings or results. One TLD badge was used for each patient.

#### 2.5.2 Dose Readings

Exposed TLD badges were sent to the Radiation Protection Institute for reading. The TLD reader system was used to process the TLD badges used to record scatter radiation. The thermoluminescent (TL) analyzer type TL1009I is suitable for research studies in radiation dosimetry [19]. When the dosimeters were loaded into the reader, it automatically heated the TL material. A photomultiplier then measured the TL‐light released during the heating of the material 9 with hot nitrogen gas from room temperature up to a maximum of 400°C. The element correction coefficient was used as a multiplier with the reader output (charge in nanocoulombs) to make the response of each dosimeter comparable to the average response of a group of calibration dosimeters. The reader calibration factor was then used to convert the raw charge data from the photomultiplier tubes (in nanocoulombs) to the dose on the TLD card. These two factors were applied as correction factors. The scatter radiation was recorded as whole‐body dose equivalent at 10 mm [H_p_(10)] and surface dose equivalent at 0.07 mm [H_p_(0.07)].

#### 2.5.3 IQ Assessment

Quantitatively, the ImageJ software version 1.54c (Wayne Rasband and Contributors, National Institutes of Health, USA) was used to assess and analyze the resulting image. The IQ parameters determined were noise and signal‐to‐noise ratio. Noise was determined as the standard deviation of the image signals obtained from ImageJ software version 1.54c (Wayne Rasband and Contributors, National Institutes of Health, USA). Conversely, the SNR was evaluated as the ratio of the mean signal intensity to the standard deviation (noise) of the signal according to Equation (2) as given by Gariani et al. [20].

(2) $$\mathrm{SNR}=\frac{\mathrm{Mean}\;\mathrm{signal}\;\mathrm{intensity}}{\mathrm{Standard}\;\mathrm{deviation}}.$$

**Figure 2:**
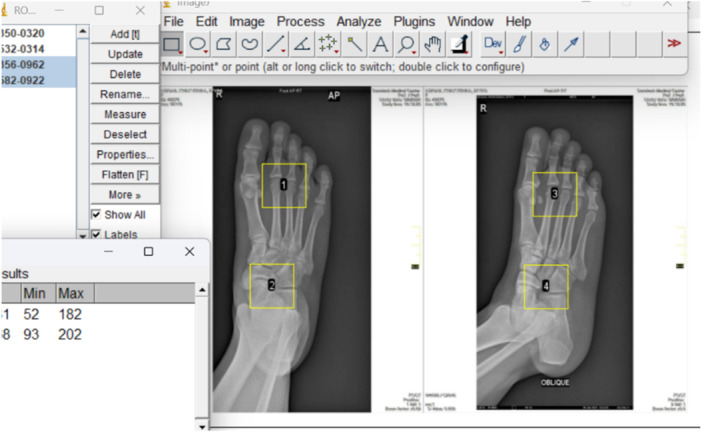
Determination of noise levels using ImageJ software.

Two square‐shaped regions of interest measuring 100 mm × 100 mm were placed at appropriate points in the image in the ImageJ (Figure 2). This was used to determine the noise level and, subsequently, the SNR. The quantitative IQ assessment was performed by the lead researcher.

### 2.6 Data Analysis

IBM SPSS Statistics v29 was used to perform both descriptive and inferential statistical analyses of the data. Descriptive statistics were used to summarize demographic characteristics. The relationship between scatter radiation and IQ was analyzed inferentially using the paired sample *t*‐test and the Spearman correlation coefficient, while the association between the effect of scatter radiation on the IQ of different body parts was assessed using the one‐way analysis of variance (ANOVA). Normality was determined with the Shapiro–Wilk test due to the small sample size.

### 2.7 Ethics Considerations

The study received ethical approval from the Ethics and Protocol Review Committee of the School of Biomedical and Allied Health Sciences, University of Ghana (Ethics approval number: SBAHS/AA/RAD/10921100/2023‐2024). In line with the Helsinki Declaration, participants provided informed consent prior to their inclusion. Additionally, to enhance anonymity and confidentiality, participants' identities were excluded.

## 3 Results

### 3.1 Demographic Data

The study included 45 patients, 60% of whom were males (*n* = 27) and 40% were females (*n* = 18). The youngest patient was 19 years old, and the oldest was 79 years old, with a mean ± standard deviation of 41.56 ± 10.40 years.

### 3.2 Exposure Factors and Measured Scatter Radiation

The basic exposure parameters measured were the tube voltage and the tube current‐time product (mAs). The mean ± standard deviation, as well as the range of exposure factors used, have been provided in Table 1.

**Table 1:** Exposure factors and measured scatter dose.

Exposure factors		
Parameter	Mean ± standard deviation	Range (minimum–maximum)
Tube voltage (kVp)	59.44 ± 7.84	50–72
Tube current‐time product (mAs)	10.64 ± 9.29	4.5–55

Measured scatter dose (Mean ± standard deviation)		
	H_p_(10) [mSv]	H_p_(0.07) [mSv]
Gender (*n*)		
Male (*n* = 27)	0.016 ± 0.0048	0.013 ± 0.0043
Female (*n* = 18)	0.018 ± 0.0024	0.019 ± 0.0031
Total (*n* = 45)	0.017 ± 0.0015	0.002 ± 0.0020
Body part (*n*)		
Shoulder/humerus (*n* = 7)	0.017 ± 0.0006	0.014 ± 0.0005
Hand/wrist/finger/forearm (*n* = 8)	0.012 ± 0.0005	0.011 ± 0.0005
femur (*n* = 6)	0.016 ± 0.0006	0.015 ± 0.0007
Knee/tibia/fibula (*n* = 17)	0.020 ± 0.0020	0.019 ± 0.0030
Foot/ankle (*n* = 7)	0.014 ± 0.0003	0.015 ± 0.0004

The scatter radiation measured as the equivalent whole‐body dose at depth of 10 mm (H_p_(10)) and surface dose at 0.07 mm (H_p_(0.07)) has been summarized in Table 1. Generally, it was observed that the scatter radiation doses were higher for females than males, albeit no significant difference from independent sample *t*‐tests (*p* = 0.72 and 0.32 for H_p_(10) and H_p_(0.07), respectively). In terms of scatter radiation produced by different body parts, the knee/tibia/fibula category produced the maximum scatter radiation, while the hand/wrist/finger/forearm group generated the least scatter radiation. However, a one‐way ANOVA test showed no significant differences between the different body parts (*F*, *p* = 0.308, 0.87; 0.223, 0.92 for H_p_(10) and H_p_(0.07), respectively). Table 1 shows a summary of the measured scattered radiation by body parts.

### 3.3 IQ

#### 3.3.1 Noise and Signal‐to‐Noise Ratio

**Figure 3:**
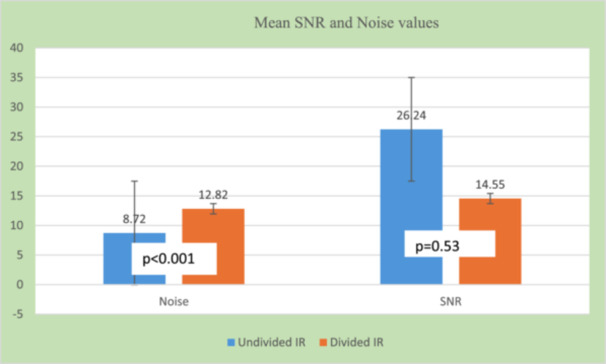
Measured noise and SNR.

The examinations performed before the image receptor (IR) was divided had a mean image noise ± standard deviation of 8.72 ± 7.14, while examinations performed after the IR was divided had a mean image noise ± standard deviation of 12.82 ± 12.19. The measured SNR of examinations performed before and after dividing the detector was 26.24 ± 12.13 and 14.55 ± 10.65, respectively. While a paired sample *t*‐test showed no significant difference in SNR of examinations performed before and after the IR was divided (*t*(44) = 0.10, *p* = 0.53), the difference between the measured noise values was statistically significant (*t*(44) = 0.51, *p* < 0.001). Figure 3 represents the mean values of the image noise and SNR measured before and after dividing the IR.

#### 3.3.2 Measured Noise Per Body Parts Before and After Dividing the IR

**Figure 4:**
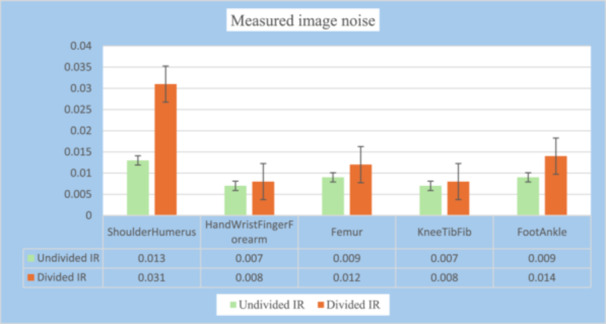
Measured image noise per body part before and after dividing the IR.

The shoulder and humerus group (*n* = 7) produced the highest image noise, while the hand, wrist, finger, and forearm group (*n* = 8) and the knee, tibia, and fibula category (*n* = 17) generated the minimum level of image noise. The noise produced from the foot and ankle group (*n* = 7) was higher than that produced by the femur category (*n* = 6). A one‐way ANOVA test showed that the differences in image noise for examinations performed prior to dividing the IR were not statistically significant between the different body parts (*F*(4) = 0.770, *p* = 0.55). However, the test revealed statistically significant differences in image noise between the body parts after dividing the IR (*F*(4) = 7.983, *p* < 0.001). Figure 4 shows a summary of the results for the noise level before and after dividing the IR.

#### 3.3.3 Measured SNR Per Body Parts Before and After Dividing the IR

**Figure 5:**
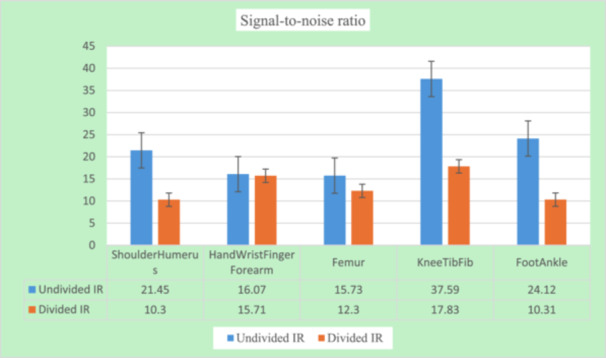
Differences in SNR between body parts.

The knee/tibia/fibula group had the highest SNR overall, although this group experienced a 47% decline in SNR. Also, the mean SNR for the shoulder and humerus group dropped from 21.45 to 10.30, resulting in a 48% decrease in SNR, the highest overall. There was approximately no change in SNR for the hand/wrist/finger/forearm group. Statistically, there were no differences in the change in SNR between the different body parts for examinations performed before and after dividing the IR (*F*, *p* = 0.0689, 0.60; 1.120, 0.36 for the divided and undivided IR, respectively). A summary of this result has been given in Figure 5.

### 3.4 Effects of the Scatter Radiation on the IQ

Although the DRT resulted in decrease in IQ (i.e., noise and SNR), Pearson correlation coefficients showed non‐significant weak positive correlations between the SNR and the dose parameters (H_p_(10) and H_p_(0.07)) and a non‐significant weak negative association between the image noise and the dose parameters (H_p_(10) and H_p_(0.07)). A summary of the Pearson correlation results has been provided in Table 2.

**Table 2:** Pearson correlation test results to determine the effect of the scatter on the image quality of examinations performed after dividing the IR.

Image quality parameter	Dose parameter	
SNR	H_p_(10)	H_p_(0.07)
Pearson *r*	0.005	0.028
Significance, *p*	0.975	0.854
Image noise		
Pearson *r*	−0.009	−0.057
Significance, *p*	0.956	0.710

## 4 Discussion

In line with the aim of the study, the discussion of this pilot study will focus on the amount of scatter radiation produced during DRTs and their effects on IQ.

### 4.1 Scatter Dose

Scatter radiation, a form of secondary radiation that deviates from its original trajectory after interacting with matter, affects the IQ and patient safety during radiographic procedures [21]. Understanding the scatter radiation dose distribution is essential for optimizing IQ [22] and is crucial for optimizing radiographic practices and improving radiation protection measures [23]. Excessive scatter radiation can reduce image contrast, making it challenging for accurate diagnosis. The analysis of scatter radiation doses measured at skin depths of 10 mm (H_p_(10)) and 0.07 mm (H_p_(0.07)) provided valuable insights into the variations and implications of scatter radiation exposure for different genders and body parts. Indeed, scatter radiation results in increased dose to surrounding persons such as patients, radiographers, and the public [1]. The amount of scatter has also been attributed to factors such as body part thickness and X‐ray beam area [1, 3, 5]. The results from Table 1 showed that the mean scatter radiation dose for females was slightly higher compared to males at both depths. This agrees with Narendran et al's [24] study on “Sex difference of radiation response in occupational and accidental exposure.” Consistent with the literature [25], although there were gender differences in scatter dose, the independent sample *t*‐tests revealed no significant statistical difference. However, the higher scatter radiation doses observed in females, although not statistically significant, may warrant additional attention in radiographic practices [26], such as tailoring imaging protocols that account for anatomical and physiological differences [27]. As seen in Table 1, the different body parts produce varying levels of scatter radiation. This is likely due to the larger volume and density of some areas compared to others, which can result in more interactions of the primary X‐ray beam with the tissues, leading to increased scatter radiation [21, 28]. For regions that produce higher scatter, such as the knee/tibia/fibula, techniques that reduce scatter, like effective collimation and the use of lower energy settings, should be considered [29].

Even though the differences in scatter radiation levels were not statistically significant, the results highlight the necessity of customized radiographic techniques and protective measures to reduce scatter radiation exposure [30]. It is crucial to ensure that radiation protection measures, such as the use of lead aprons and thyroid shields, are adequately implemented to minimize exposure, especially for more sensitive populations [31], like pediatrics. Considering that scatter radiation contributes to the overall radiation risks, strategies to reduce scatter, such as collimation and the use of anti‐scatter grids, should be emphasized during radiographic procedures, and scatter correction methods can also be employed [1]. The findings underscore the need for continuous monitoring and optimization of radiographic practices to ensure patient safety and IQ. By addressing the factors contributing to scatter radiation production and implementing effective protective measures, radiographic professionals can enhance the diagnostic efficacy and safety of radiographic examinations [32].

### 4.2 Effects of Scatter Radiation on IQ

The noise and SNR are essential parameters for determining the diagnostic value of radiographic images [33]. The analysis showed a statistically significant increase in noise after the IR was divided (Figure 3). This is consistent with Sayed et al. [1], who indicated that scatter may result in a non‐diagnostic image due to an increase in noise production. Additionally, Aichinger et al. [2] study reported that scatter radiations negatively affect the IQ in terms of image artifacts and reduced image contrast. The findings could be attributed to factors such as the reduction in the effective area of the IR, which could lead to increased scatter radiation and ineffective absorption of primary radiation [34]. Additionally, the DRT resulted in a non‐significant decrease in SNR (Figure 3). The Pearson correlation test showed a non‐significant influence of scatter radiation on IQ in the context of this study (Table 2), potentially due to effective scatter control mechanisms in place. However, the findings have important implications for radiographic practice. Specifically, high noise levels can obscure fine details and subtle differences in tissue contrast, making it more challenging to identify pathologies, while lower SNR further exacerbates this issue by reducing the overall diagnosability of the image [35]. Moreover, previous studies have reported that forward‐scattered X‐rays degrade radiographic IQ [5, 6, 7] and are therefore unacceptable in radiographic imaging [1]. To mitigate the adverse effects on IQ, it may be necessary to adjust imaging parameters, such as increasing exposure settings, to compensate for the higher noise levels and reduced SNR. However, this must be done with caution to avoid an unnecessary increase in patient dose. Other documented strategies to mitigate the effects of scatter radiation include air gaps, aperture diaphragms, and digital image processing [16].

### 4.3 Limitations

The study used a relatively low sample size, which could limit the generalizability of the results. Therefore, interpretation of the findings should be made with caution. Additionally, the study did not investigate the influence of factors such as tube voltage, tube current‐time product, and cast on the dose and IQ, while the influence of the sample size was also not accounted for. However, the pre‐examination quality assurance procedures helped to obtain accurate and reliable results. The collimation distance was also greater than 1 cm, which may limit the implications of the findings. As a pilot study, we advise future studies to consider all confounding variables and to use a larger sample to provide more generalizable results.

## 5 Conclusion

The impact of scatter radiation on IQ is a critical consideration in radiographic imaging, particularly when evaluating the performance of different imaging techniques and exposure settings. This study revealed significant differences in noise and non‐significant differences in SNR, although the differences did not result from the scatter dose produced from the DRT during extremity radiography. Regardless, the results call for the need for radiographers to adopt strategies to further reduce the associated scatter radiation, as these have detrimental effects on both patients and professionals. These strategies may include the use of anti‐scatter grids, effective collimation, and radiation dose optimization training for radiographers. Further investigation into the factors contributing to the observed differences is recommended.

## Author Contributions

**Erica Mawusinu Domi:** conceptualization, writing – original draft, methodology, visualization, writing – review and editing, formal analysis. **Jacob Leonard Ago:** conceptualization, writing – original draft, methodology, visualization, writing – review and editing, formal analysis. **William K. Antwi:** writing – review and editing, supervision. **Derick Seyram Sule:** writing – review and editing, supervision. **Godwill Acquah:** writing – review and editing, methodology. **Godsway Yao Gagoh:** conceptualization, writing – review and editing. **Ophelia Andowah Otsiwah:** writing – review and editing. All authors have read and approved the final version of the manuscript. Jacob Leonard Ago had full access to all of the data in this study and takes complete responsibility for the integrity of the data and the accuracy of the data analysis.

## Funding

The authors have nothing to report.

## Ethics Statement

The work was ethically approved by the Ethics and Protocol Review Committee of the School of Biomedical and Allied Health Sciences, University of Ghana (SBAHS/AA/RAD/10921100/2023‐2024). In line with the Helsinki Declaration, participants provided informed consent prior to their inclusion. Additionally, to enhance anonymity and confidentiality, participants' personal identities were not included.

## Consent

The authors have nothing to report.

## Conflicts of Interest

The authors declare no conflicts of interest.

## Transparency Statement

The lead author, Jacob Leonard Ago, affirms that this manuscript is an honest, accurate, and transparent account of the study being reported; that no important aspects of the study have been omitted; and that any discrepancies from the study as planned (and, if relevant, registered) have been explained.

## Supporting information


**Supporting File:** hsr272353‐sup‐0001‐Supplementary_sheet.docx.

## Data Availability

The data associated with this may be made available by the corresponding author upon reasonable request.
