# Supplementary material for: Quantitative Assessment of the Effects of Scatter Radiation on Image Quality During Extremity Radiography at a Leading Tertiary Hospital in Ghana: An Experimental Pilot Study
Source: Health Sci Rep. 2026 Apr 16;9(4):e72353. doi: 10.1002/hsr2.72353 (PMC13087104; doi:10.1002/hsr2.72353)
Supplement: Supplementary file 1 — Supporting File: hsr272353‐sup‐0001‐Supplementary_sheet.docx. [file HSR2-9-e72353-s001.docx]

**Supplementary sheets**

Supplementary sheet 1: Data collection sheet

| **ID** | **PROCEDURE** | **GENDER** | **AGE** | **KVp** | **mAs** | **ECC** | **RCF** |
| --- | --- | --- | --- | --- | --- | --- | --- |
|  |  |  |  |  |  |  |  |
|  |  |  |  |  |  |  |  |
|  |  |  |  |  |  |  |  |
|  |  |  |  |  |  |  |  |
|  |  |  |  |  |  |  |  |
|  |  |  |  |  |  |  |  |
|  |  |  |  |  |  |  |  |
|  |  |  |  |  |  |  |  |

Supplementary sheet 2


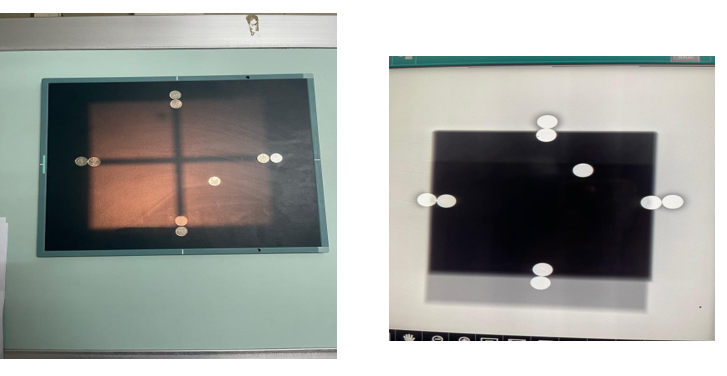


Set up for QC test. The two images above show a picture of the light and radiation field congruence test and its resulting image.

Supplementary sheet 3: Procedure for dose measurement


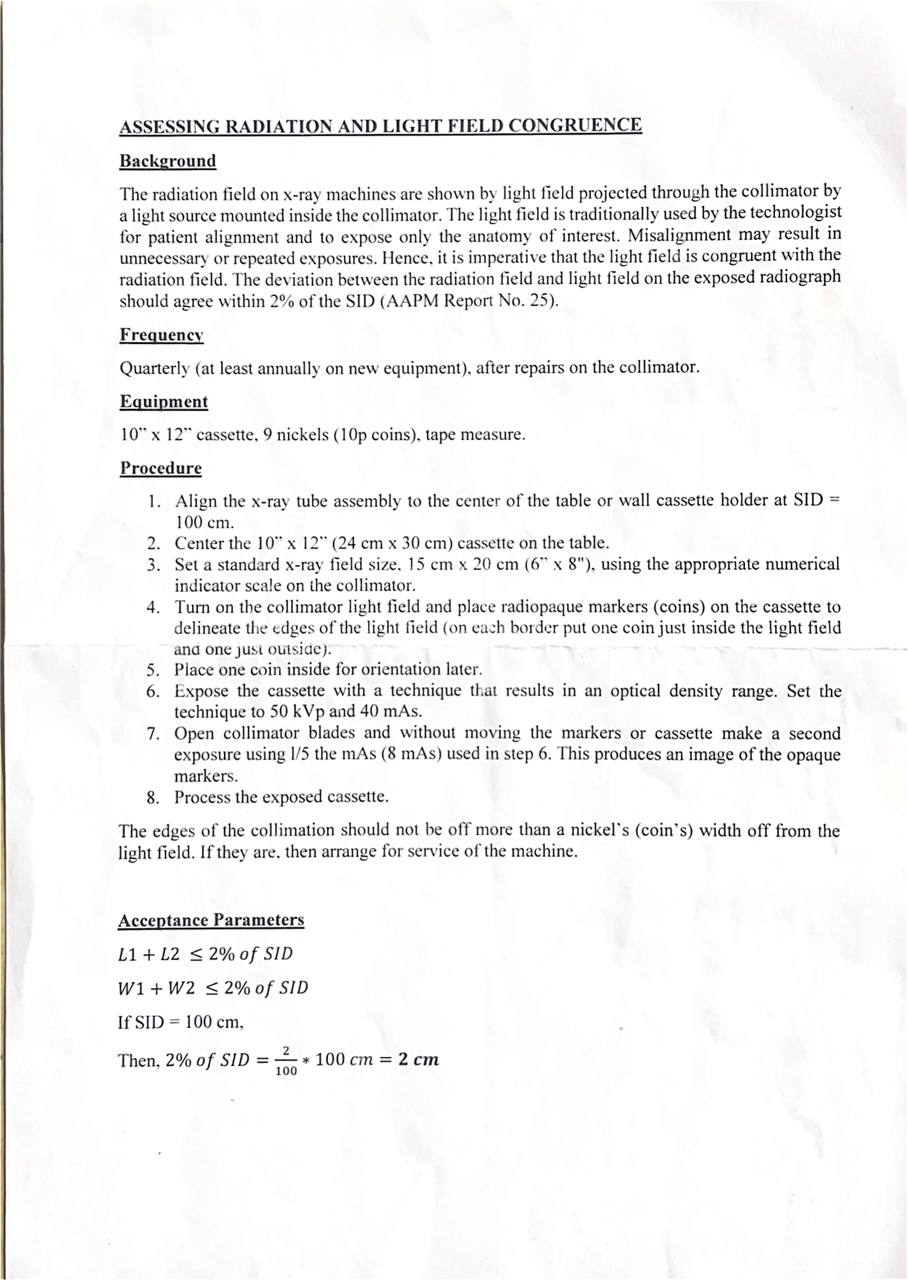


Supplementary sheet 4: Sample dose readings

**
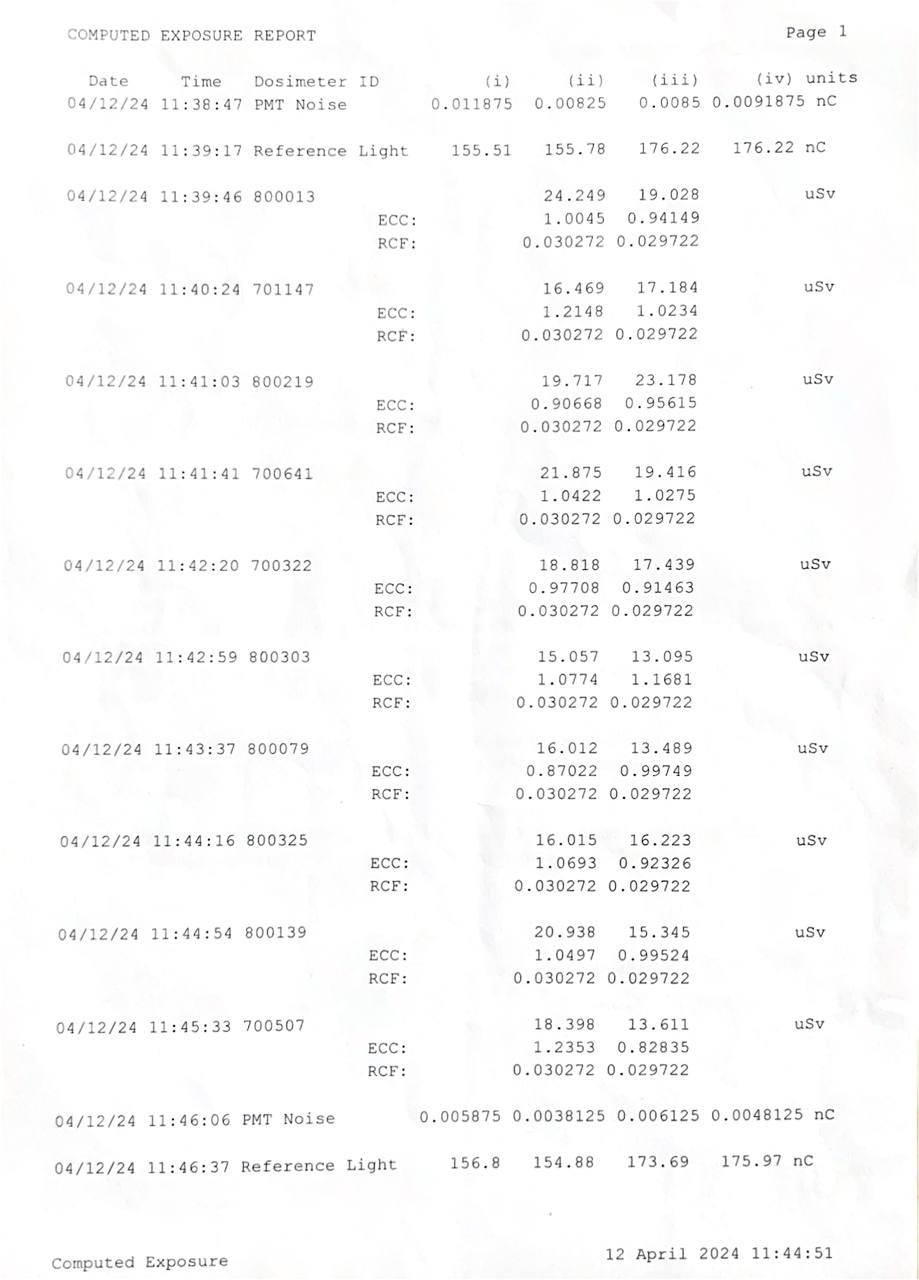
**
